# Integrated Single-Tip IMAC-HILIC Enables Simultaneous Analysis of Plant Phosphoproteomics and N‑Glycoproteomics

**DOI:** 10.1021/acs.jproteome.5c00185

**Published:** 2025-06-23

**Authors:** Chin-Wen Chen, Ting-An Chen, Pei-Yi Lin, Shu-Yu Lin, Chuan-Chih Hsu

**Affiliations:** † 71559 Institution of Plant and Microbial Biology, Academia Sinica, Taipei 115201, Taiwan; ‡ Academia Sinica Common Mass Spectrometry Facilities for Proteomics and Protein Modification Analysis, Academia Sinica, Taipei 115201, Taiwan

**Keywords:** phosphoproteomics, N-glycoproteomics, enrichment, immobilized metal affinity chromatography, hydrophilic interaction Chromatography

## Abstract

Protein phosphorylation and N-glycosylation are key post-translational modifications (PTMs) in plants that regulate critical signaling processes. However, coanalysis of these PTMs is often complicated by their relatively low abundance and divergent enrichment requirements. Here, we present a single-tip IMAC-HILIC approach that integrates immobilized metal affinity chromatography (IMAC) and hydrophilic interaction chromatography (HILIC) materials within one pipet tip, enabling concurrent enrichment and sequential elution of phosphopeptides and N-glycopeptides. This integrated workflow effectively reduces phosphopeptide coelution during N-glycopeptide elution and streamlines sample processing. In direct comparison with the tandem-tip TIMAHAC method, our single-tip strategy achieves a comparable identification depth and offers superior quantitative accuracy for N-glycopeptides. We further demonstrate its applicability by examining the impact of calcium deprivation in *Arabidopsis*, revealing distinct global changes in both the phosphoproteome and N-glycoproteome. Our optimized protocol thus provides a straightforward and high-throughput platform for dual PTM profiling in complex plant samples, paving the way for broader investigations of PTM crosstalk in diverse physiological and stress responses.

## Graphical abstract

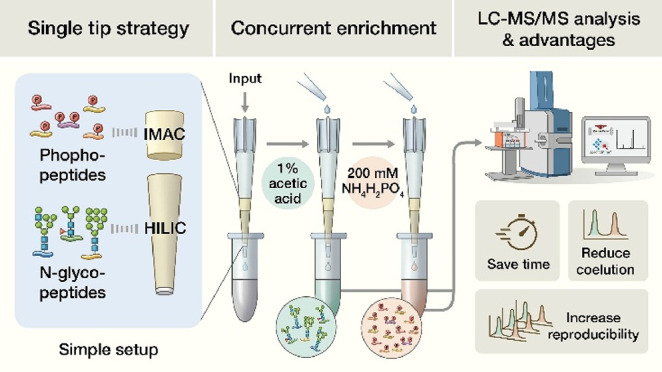

## Introduction

Post-translational modifications (PTMs) play essential roles in plant cells by sensing and transducing diverse environmental cues to downstream targets.^1–4^ Among these PTMs, protein phosphorylation and N-glycosylation are particularly critical in early recognition events of extracellular signals.^5–8^ This is exemplified by receptor kinases (RKs) such as MIK2, BAK1, and EFR, which reside at the plasma membrane to regulate plant immune responses or growth pathways and are modified by both PTMs.^9–12^ However, whether these two modifications have synergistic effects on RK activation and fine-tuning of downstream signaling remains unclear. A systematic examination of how the plant phosphoproteome and N-glycoproteome are altered under environmental perturbations could illuminate potential crosstalk between these PTMs and their complex regulatory coordination.

Despite advances in mass spectrometers, global detecting phosphopeptides and N-glycopeptides remains challenging due to their comparatively low abundance and ionization efficiency.^13,14^ Consequently, well-established enrichment strategies are routinely employed before mass spectrometry (MS) analysis.^15,16^ For phosphopeptides, methods such as immobilized metal affinity chromatography (IMAC) and metal oxide affinity chromatography (MOAC) typically leverage electrostatic interactions between metal ions or metal oxides and the negatively charged phosphate groups.^17–20^ In contrast, the separation of intact N-glycopeptides from nonglycosylated peptides primarily exploits the hydrophilic properties of the attached glycans, with hydrophilic interaction chromatography (HILIC) and mixed-mode strong anion exchange (MAX) serving as two principal approaches in glycoproteomics.^21–24^ However, simultaneous monitoring of the phosphoproteome and N-glycoproteome in complex biological samples remains difficult, largely due to the differing materials and protocols needed for each method, which limit throughput and reproducibility.

To simplify and enhance the accuracy of dual PTM analyses, several strategies have been developed to enable simultaneous phosphopeptide and N-glycopeptide enrichment within a single workflow.^25–29^ These approaches employ materials with dual-mode affinity—combining hydrophilic and electrostatic interactions—to capture both modified peptide types.^30–33^ However, the fabrication of certain materials can be complex, limiting accessibility to the broader proteomics community, and their scalability is sometimes unproven when only standard proteins are used for demonstration. Additionally, coelution of PTM-modified peptides in each fraction increases sample complexity, compromising MS analytical depth and quantitative performance. To address these limitations, we previously introduced a tandem S-Trap-IMAC-HILIC (TIMAHAC) approach for concurrent plant phosphoproteomics and N-glycoproteomics.^34^ In TIMAHAC, we demonstrated that a unified loading buffer can be used for both IMAC and HILIC with a high enrichment selectivity. This success prompted us to explore integrating both materials into a single pipet tip for concurrent enrichment, aiming to eliminate the need for a tandem-tip format while further improving simplicity, reproducibility, and throughput.

Here, we present a streamlined, high-throughput enrichment approach that combines IMAC and HILIC materials in a single pipet tip for concurrent phosphopeptide and N-glycopeptide enrichment, followed by sequential elution. In addition to its simplified operating procedure, the single-tip format improves reproducibility over tandem-tip setups, as demonstrated by direct benchmarking against our established TIMAHAC method. We further show the utility of this approach by investigating phosphorylation–N-glycosylation crosstalk in *Arabidopsis* under calcium deprivation. Overall, this method provides a robust platform for high-throughput PTM profiling, advancing our understanding of plant signaling networks.

## Methods

### Supporting Information

The details of chemicals and materials, plant culture and treatment, protein lysis and digestion,^35^ IMAC and HILIC enrichment,^34,36^ and LC–MS/MS analysis are provided in the Supporting Information.

### IMAC-HILIC Tip Enrichment

IMAC-HILIC tip enrichment was performed by combining the C8-based HILIC tip with Fe-NTA silica beads with the following modifications. Briefly, after generating the C8 tip and packing it with HILIC beads, 5 mg of equilibrated Fe^3+^-NTA beads (in 200 μL of 1% (v/v) TFA, 80% (v/v) ACN) was transferred into the HILIC-C8 tip by centrifugation at 1000*g* for 5 min).

Tryptic peptides eluted from the S-Trap microcolumn were loaded into the IMAC-HILIC tip by tandem-tip centrifugation at 1000*g* for 5 min. The tip was then washed twice with 100 μL of 1% (v/v) TFA in 80% (v/v) ACN (1000*g*, 5 min each). Next, the IMAC-HILIC tip was inserted into an activated Evotip, and the N-glycopeptides were eluted into the Evotip using 100 μL of either 1% (v/v) AA, 0.5% (v/v) TFA, or 0.5% (v/v) FA (1000*g*, 5 min), depending on the experimental comparison. Phosphopeptides were subsequently eluted into a microcentrifuge tube using 100 μL of 200 mM NH_4_H_2_PO_4_, followed by 1% (v/v) TFA in 80% (v/v) ACN (1000g, 5 min). The eluate was then lyophilized, resuspended in 50 μL of 0.1% (v/v) TFA, and loaded into an activated Evotip for LC–MS/MS analysis.

### Data Processing

For phosphoproteomics analyses, raw MS files were searched against the Araport11 database (48,266 entries, release date: 2022-09-15; downloaded from TAIR Web site, https://www.arabidopsis.org/) using SpectroMine (version 4.5, Biognosys). Trypsin was specified as the proteolytic enzyme, allowing for up to two missed cleavages. The fixed modification was set as carbamidomethyl (C), and variable modifications were set as oxidation (M), acetylation (protein N-term), and phosphorylation (STY). The MS tolerance and false discovery rate (FDR) were maintained at the default software settings. PTM localization probability cutoff was set to zero for data completeness. A list of localized phosphorylation site was generated using Peptide Collapse (version 1.4.4)^37^ with a localization cutoff of 0.75 (class I sites).

For N-glycoproteomics analyses, raw MS files were searched against the Araport11 database (96,532 entries, 50% decoys) using MSFragger^38^ (version 4.1) within the FragPipe platform^39^ (version 22.0). The glyco-N-LFQ workflow was employed for the downstream processing. Variable modifications included the STY site with 79.96633 mass delta, and N-glycan searches were performed against the N-glycan 52 plants database from Byonic. All other search parameters were maintained in their default settings.

### Data Analysis

The number of unique peptides and phosphopeptides identified in each sample was determined using SpectroMine output reports, excluding phosphopeptides with intensities reported as “NaN”. Unique N-glycopeptides in each sample were quantified using the combine_modified_peptide table generated by FragPipe. The accumulated extracted ion chromatogram (XIC) area was calculated by summing the intensities of all identified phosphopeptides and N-glycopeptides. Error bars in the histogram indicate the standard deviation.

For boxplots, the lower and upper hinges indicate the first and third quartiles, respectively, with the central bar representing the median. The upper and lower whiskers extend to the highest and lowest values within the 1.5 interquartile range. Coefficient of variation (CV) values were computed from peptides detected in all three replicates. The results were visualized using GraphPad Prism (version 10).

N-Glycopeptides were classified into five glycosylation-type categories based on their glycan composition: truncated (1–2 HexNAc), paucimannose (2 HexNAc and 1–3 Hex), high mannose (2 HexNAc and >3 Hex), hybrid (3 HexNAc and 3–5 Hex), and complex (4 HexNAc and 3–5 Hex). Paucimannose, high mannose, hybrid, and complex N-glycans may include fucose (Fuc) or xylose (Pent).

Quantitative analyses were conducted using Perseus^40^ (version 2.0.7.0). Phosphorylation sites and glycopeptides were considered quantifiable if identified in at least three replicates. Intensities were log2-transformed and replaced by NA if the transformed values less than 3. Missing values were imputed from a normal distribution (with = 0.2, down shift = 2.0). A two-sample Student’s *t* test was employed to identify significantly altered phosphorylation sites and glycopeptides, using a permutation-based false discovery rate (FDR) threshold of 0.05 and a minimum 2-fold change. Significantly changing sites and glycopeptides were z-scored and subjected to hierarchical clustering analysis. Gene ontology (GO) enrichment analysis was performed using Fisher’s exact test (*p* < 0.05, fold enrichment >1), through the PANTHER database.^41^ The grand average of hydropathy (GRAVY) value for peptide sequences was calculated using the Web site: https://www.gravy-calculator.de/.

## Results and Discussion

### Design of a Single IMAC-HILIC Enrichment Tip

We previously developed the TIMAHAC method utilizing a tandem arrangement of IMAC and HILIC tips to concurrently enrich both phosphopeptides and N-glycopeptides. Because both of separation methods employ the same peptide loading buffer, we investigated whether placing both types of beads within a single pipet tip (IMAC-HILIC tip) could further simplify the workflow while maintaining enrichment efficiency and selectivity.

In the designed IMAC-HILIC tip, IMAC beads are placed at the top, followed by HILIC beads and finally a C8 disk at the bottom, serving as a frit (Figure S1). This specific arrangement follows our previous findings that tandem IMAC-HILIC enrichment outperforms the reversed order.^34^ By consolidating the two types of beads into a single tip, we eliminate the need to prepare separate chromatography tips and individually elute each PTM. Instead, enriched phosphopeptides and N-glycopeptides can be sequentially recovered in two fractions: first, by eluting N-glycopeptides through disrupting their hydrophilic interactions with the HILIC layer and subsequently releasing phosphopeptides by breaking their electrostatic interactions with Fe^3+^ ions (Figure 1).

**Figure 1:**
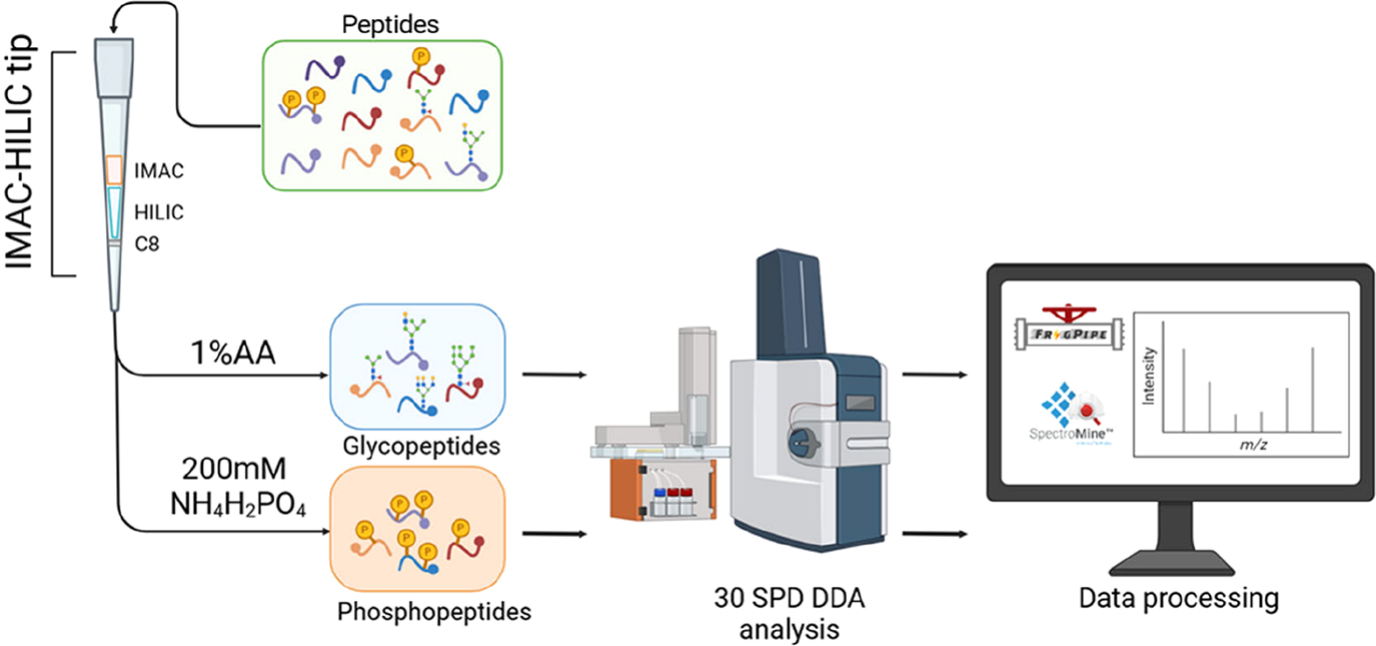
A single IMAC-HILIC tip serves as a unified platform for the simultaneous enrichment of phosphopeptides and N-glycopeptides. Contaminant removal and proteolysis are performed using an S-Trap microcolumn. Digested peptides are eluted with 80%ACN/1%TFA into the IMAC-HILIC tip via centrifugation. Within the tip, N-glycopeptides and phosphopeptides are captured and subsequently eluted sequentially using 1% AA and ammonium phosphate solution, respectively. The enriched peptides are analyzed using an Evosep system coupled to a timsTOF HT mass spectrometer in the 30 SPD DDA mode. Phosphoproteomics and N-glycoproteomics data are processed with SpectroMine and FragPipe, respectively.

Efficient separation of phosphopeptides and N-glycopeptides in a single-tip workflow is critical for achieving high sensitivity and broad coverage in PTM identification.^27^ Typically, aqueous solutions with organic acids (e.g., FA and TFA) are used to release N-glycopeptides from the HILIC stationary phase.^42^ However, low pH values and the competition interactions of these acids can disrupt the electrostatic binding between phosphopeptides and Fe^3+^ ions,^43,44^ leading to undesired coelution in the HILIC fraction. As a result, optimizing the HILIC elution buffer composition is a key consideration in developing a single IMAC-HILIC tip strategy that achieves effective separation while maintaining the overall enrichment efficiency.

### Impact of HILIC Elution Conditions on Phosphopeptide Retention

To assess the degree of phosphopeptide coelution, HILIC elution buffer containing 0.5% FA (pH = 2.5) and 0.5% TFA (pH = 1.5) was used as the first elution fraction and compared against 1% AA (pH = 2.6), employed as an IMAC washing buffer. Subsequently, phosphopeptides retained on IMAC were eluted with an ammonium phosphate solution (second elution). The results revealed that 52% and 41% of the peptides identified in the TFA and FA fraction, respectively, were phosphorylated—predominately monophosphorylated peptides—whereas only 18% were phosphorylated in the AA fraction (Figure 2A; Table S1A–C). A similar trend was observed when comparing accumulated phosphopeptide intensities (Figure S2A). As expected, the second elution (ammonium phosphate) exhibited a substantial decrease in monophosphorylated peptide identifications for the TFA and FA fractions relative to standard IMAC washing with 1% AA (Figure 2B; Table S1D–F). Moreover, two-thirds of the phosphopeptides identified in the first AA elution were also identified in the second elution, contrasting with only 13% and 28% overlap for TFA and FA, respectively (Figure S2B). These findings indicate that AA only minimally displaced phosphopeptides from Fe^3+^-IMAC compared with TFA or FA.

**Figure 2:**
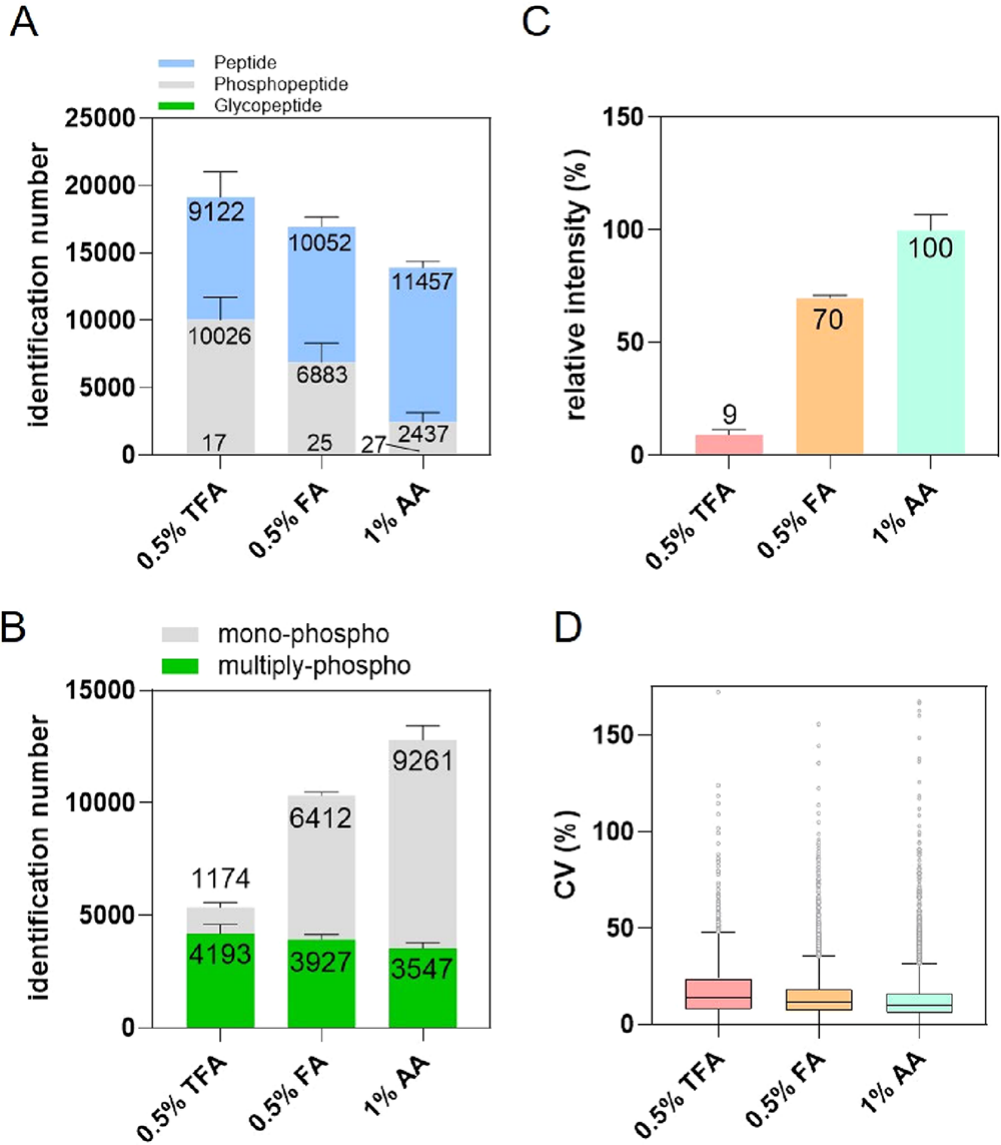
Evaluation of organic acids in the HILIC elution buffer on phosphopeptide retention using Fe^3+^-IMAC. (A) Number of unique peptides, phosphopeptides, and N-glycopeptides identified in the first elution fraction using three different organic acids: AA, TFA, and FA. (B) Number of unique monophosphorylated and multiply phosphorylated peptides in the second elution with ammonium phosphate following the first elution with AA, TFA, and FA. (C) Accumulated XIC area of monophosphorylated peptides identified in the second elution, expressed relative to the total intensity for the AA condition (set to 100%). (D) Boxplot illustrating the distribution of the CVs for phosphopeptides identified in the second elution following the first elution with AA, TFA, and FA.

Quantitative analyses of MS1 intensities revealed that monophosphorylated peptide signals were reduced 91% in the TFA condition and 30% in the FA condition during the second elution (Figure 2C). Correspondingly, the median log2 intensities for these fractions were 1.31-fold and 1.02-fold lower, respectively, than those obtained from standard IMAC enrichment (1% AA) (Figure S2C). Notably, the mild coelution observed in the AA condition contributed to the lowest replicate variation (CV = 10.3%) among the three conditions (Figure 2D). Overall, these results demonstrate that eluting with TFA or FA can induce substantial coelution of monophosphorylated peptides, thereby reducing the reproducibility in Fe^3+^-IMAC elution. Consequently, we hypothesize that AA-based solution may serve as a more selective HILIC elution buffer to minimize phosphopeptide coelution.

### Comparison of Organic Acids for HILIC N-Glycopeptide Elution Efficiency

To further investigate the potential of the AA solution, we evaluated its performance in releasing N-glycopeptides from HILIC alone compared to conventional elution buffers (0.5% TFA and 0.5% FA). Enrichment efficiency was assessed on the basis of the depth of N-glycopeptide identification and the glycan distribution among the identified peptides.

Using the 1% AA solution, an average of 2617 unique N-glycopeptides per replicate was identified with 3922 unique N-glycopeptides combined across all three replicates (Figure 3A; Table S2). Notably, the AA condition resulted in fewer coeluted phosphopeptides, averaging 20% and 23% less than the FA and TFA conditions, respectively, consistent with the previous IMAC coelution results. Despite a slightly lower identification depth (9% fewer glycopeptides count-based and 8% less intensity-based on average) compared to TFA elution, AA elution achieved the same median log2MS1 intensities (Figures 3B and S3A). Importantly, of the 3922 N-glycopeptides identified using AA elution, 3469 (88%) overlapped with those identified by FA or TFA elution (Figure S3B). Further analysis of glycan structure distributions revealed no significant differences among the three elution conditions (Figure S3C).

**Figure 3:**
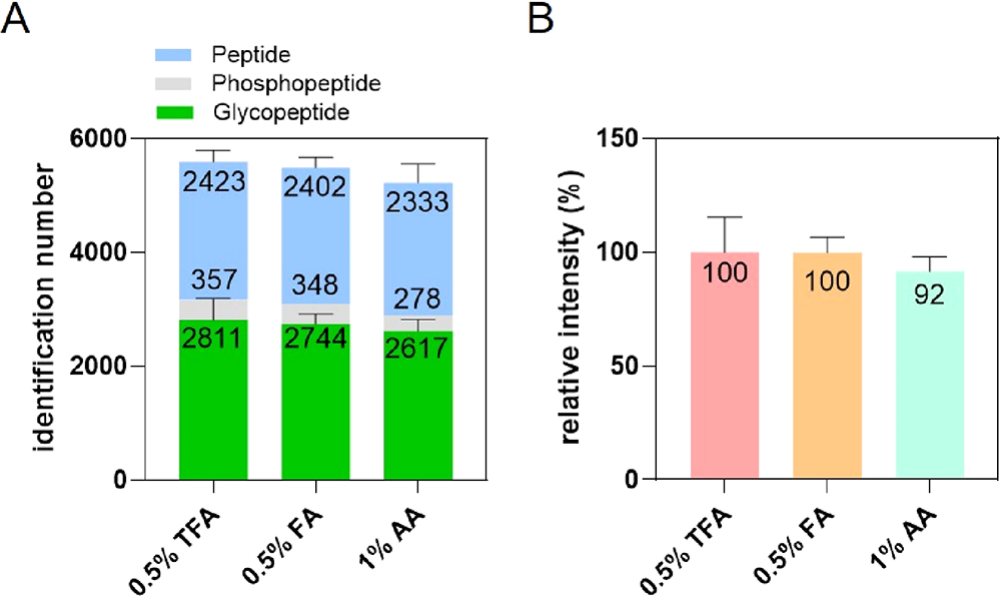
Assessment of the N-glycopeptide elution efficiency using three organic acids in HILIC. (A) Number of unique peptides, N-glycopeptides, and phosphopeptides identified after elution with AA, TFA, and FA. (B) Accumulated XIC area of identified N-glycopeptides for each organic acid, with the total intensity for TFA set to 100%.

Collectively, these results suggest that the AA solution achieves moderate elution efficiency relative to that of TFA and FA, with comparable N-glycopeptide intensities and similar glycan structural coverage. Crucially, the AA condition significantly reduces phosphopeptide coelution in HILIC, which was a major drawback observed with FA and TFA elution buffers. Therefore, we propose 1% AA as the optimal HILIC elution buffer for releasing N-glycopeptides in this IMAC-HILIC workflow, balancing both the glycopeptide yield and phosphopeptide selectivity.

### Assessing the Enrichment and Separation Efficiency of the IMAC-HILIC Tip

After establishing an optimal elution strategy for both phosphopeptides (PPs) and N-glycopeptides (GPs) using the IMAC-HILIC tip, we next evaluated the enrichment depth, reproducibility, and separation efficiency. Initial GP fractions were eluted using TFA, FA, or AA, followed by elution of the PP fraction. Elution of the GP fraction with AA significantly minimized phosphopeptide coelution compared to FA and especially TFA (Figure 4A). This reduction in phosphopeptide contamination within the GP fraction correspondingly enhanced N-glycopeptide identification. Notably, this improvement occurred despite AA yielding fewer N-glycopeptides in a standalone HILIC enrichment compared to TFA and FA (Figure 3A). This suggests that reduced sample complexity, achieved by minimizing phosphopeptide coelution, is a key determinant for improving N-glycopeptide detection depth in the integrated IMAC-HILC approach.

**Figure 4:**
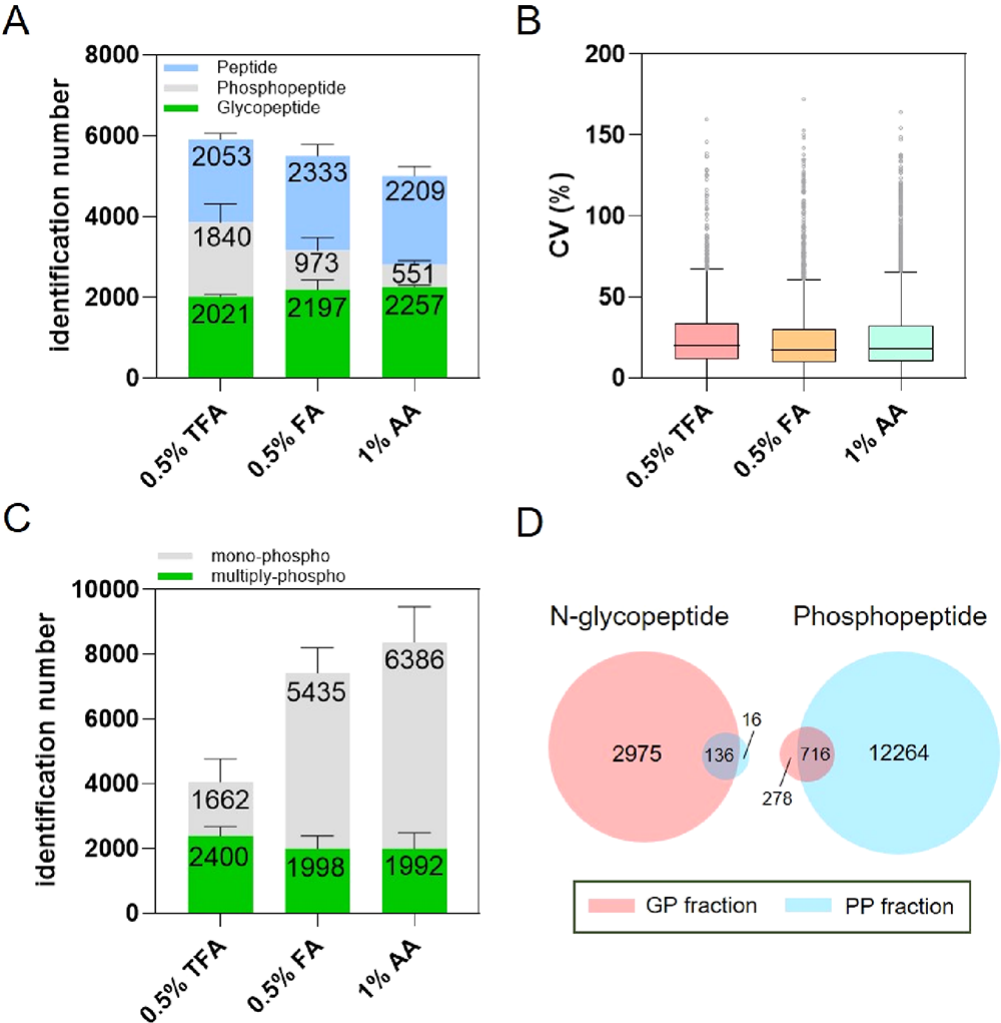
Impact of coelution on peptide identification and reproducibility of the two IMAC-HILIC fractions. (A) Number of unique peptides, phosphopeptides, and N-glycopeptides identified in the GP fraction after its elution with three acids: AA, TFA, and FA. (B) Boxplot illustrating the distribution of CVs for N-glycopeptides identified within the GP fraction when eluted with AA, TFA, and FA. (C) Number of unique monophosphorylated and multiply phosphorylated peptides identified in the subsequent PP fraction following the initial GP fraction elution with AA, TFA, and FA. (D) Overlap analysis of identified N-glycopeptides (left) and phosphopeptides (right) between the GP and PP fractions when AA was utilized as the elution buffer for the GP fraction.

Additionally, the improved separation with AA enhanced the reproducibility of N-glycopeptide quantification, evidenced by a lower median CV of 17.7% for N-glycopeptides, compared to 19.9% under the high coelution of TFA condition (Figure 4B). Consistent with the improved phosphopeptide retention during GP elution, using AA for the initial GP fraction resulted in the highest number of monophosphorylated peptides identified in the subsequent PP fraction, significantly more than when using TFA or FA for GP elution (Figure 4C).

Separation efficiency was assessed by the overlap of phospho- and N-glycopeptides between the PP and GP fractions. When AA was used for GP elution, only 7.5% of total identified phosphopeptides (994 unique phosphopeptides) were detected in the GP fraction, indicating minimal coelution (Figure 4D). More than 99% of N-glycopeptides were recovered in the GP fraction regardless of the acid used (AA, FA, or TFA), suggesting that the specific type and concentration of organic acid used as the mobile phase did not substantially impact N-glycopeptide elution (Figures 4D and S4). Collectively, these data demonstrate that phosphopeptide coelution into the GP fraction negatively impacts N-glycopeptide identification depth and reproducibility. AA is therefore the superior eluent for the GP fraction in this single-tip enrichment strategy, enabling optimal separation and maximizing N-glycopeptide coverage.

### Benchmarking the IMAC-HILIC Tip against the TIMAHAC Approach

To compare the performance of the IMAC-HILIC tip with that of a tandem enrichment approach, we benchmarked it against our previously reported TIMAHAC method in terms of identification depth and reproducibility. At first glance, IMAC-HILIC recovered fewer phospho- and N-glycopeptides than TIMAHAC (Figure S5A; Table S3). However, the IMAC-HILIC tip outperformed the TIMAHAC method in quantitative accuracy, as evidenced by lower median CV values for both phosphopeptides and N-glycopeptides detected in all technical replicates (Figure S5B,C). Notably, IMAC-HILIC tip demonstrated a marked advantage in reproducibility for N-glycopeptides (16.7% CV vs 25.3% CV), likely because the single-tip design avoids the additional variability introduced by the second tip in the tandem format.

When examining the proportion of peptides with CV values <20%, IMAC-HILIC showed more identification number and superior reproducibility for quantifiable N-glycopeptides, surpassing TIMAHAC despite identifying fewer total N-glycopeptides (Figures S5A and 5A). For phosphopeptides, although a higher percentage achieved CV values below 10% and 20% with IMAC-HILC, the number was still approximately 10% lower than that in TIMAHAC. This outcome may stem from the C8 disk frit used in the single-tip format, which necessitates an organic solvent elution and can lead to additional phosphopeptide loss (Figure 5B).

**Figure 5:**
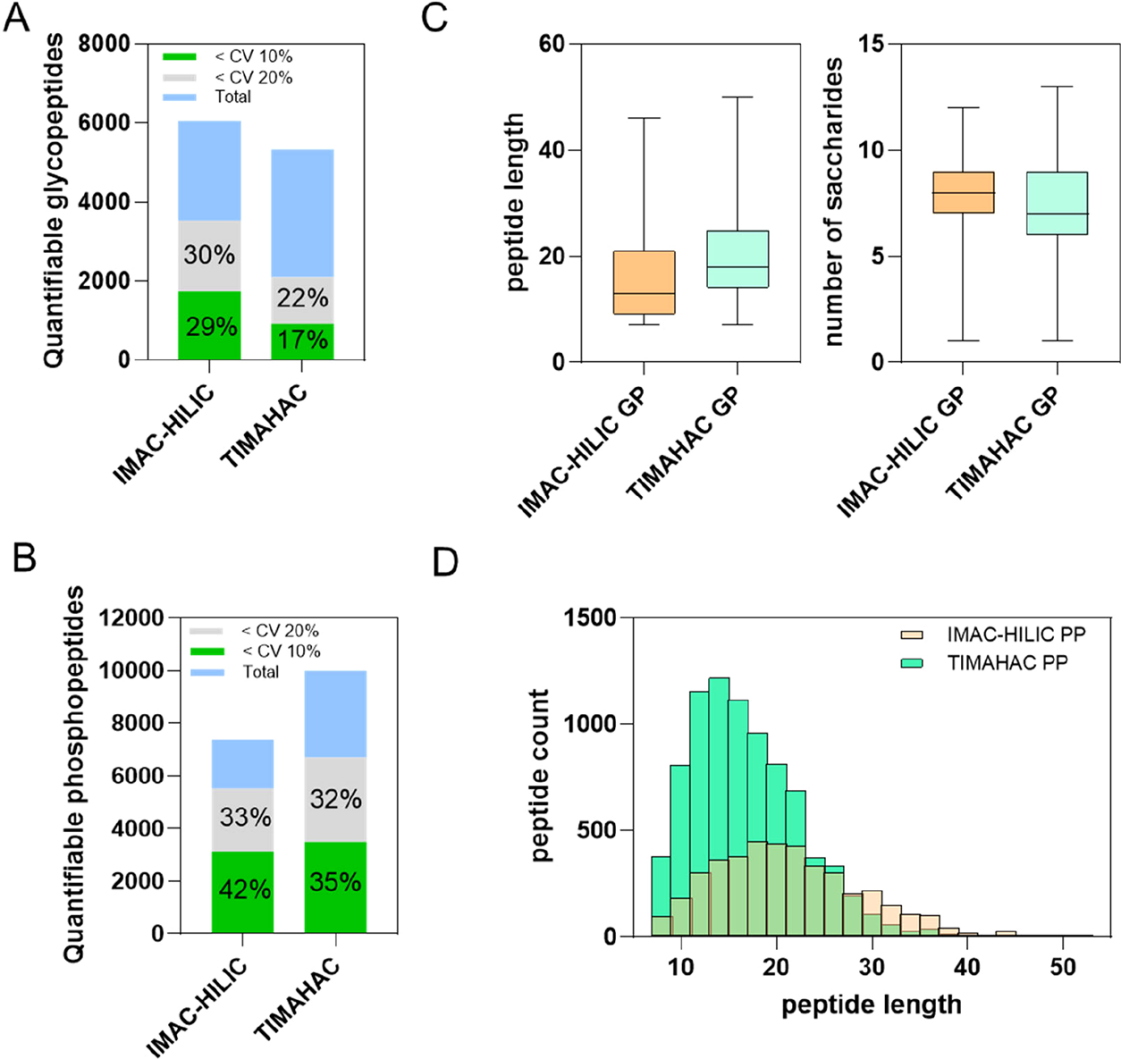
Benchmarking the quantitative performance of the IMAC-HILIC tip against the TIMAHAC approach. Comparison of the number and percentage of quantifiable (A) N-glycopeptides and (B) phosphopeptides with CVs below 10% and 20%. Peptides were considered quantifiable if they were identified in at least two out of three technical replicates. (C) Distributions of peptide length and glycan saccharides count for N-glycopeptides identified using each method. (D) Histogram showing the distribution of phosphopeptide lengths in both approaches.

Each strategy favored different subsets of N-glycopeptides and phosphopeptides (Figure S5D). Unique N-glycopeptides identified by IMAC-HILIC tended to be shorter and contained more saccharides than those unique to TIMAHAC (Figure 5C). Additionally, lower median GRAVY values indicate greater hydrophilicity (Figure S5E). By contrast, phosphopeptides uniquely identified by IMAC-HILIC were generally longer and more hydrophobic than those unique to TIMAHAC (Figures 5D and S5F). Overall, these results highlight the reproducibility advantage offered by the single-tip IMAC-HILIC approach over the tandem TIMAHAC enrichment as well as unique enrichment biases inherent to each method. We anticipate that the IMAC-HILIC tip will provide a robust platform for studying the crosstalk and remodeling of protein phosphorylation and N-glycosylation in plant responses to environmental stimuli.

### IMAC-HILIC Delineates the Impact of Calcium Deprivation on *Arabidopsis* Phosphoproteome and N-Glycoproteome

Fluctuations in cytosolic-free calcium concentration are critical biochemical events in plant osmotic stress responses, triggering the activation of protein kinases and subsequent downstream signaling.^45^ Our previous work revealed that treating *Arabidopsis* seedlings with EGTA, a calcium chelator, induces activation of the RAF-SnRK2 cascade in a manner similar to mannitol treatment, which chemically mimics osmotic stress.^5^ However, how calcium deprivation affects the plant N-glycoproteome and the extent of crosstalk between phosphorylation and N-glycosylation remains unclear.

Leveraging our IMAC-HILIC tip for concurrent phosphopeptide and N-glycopeptide enrichment, we profiled the global proteome, phosphoproteome, and N-glycoproteome in *Arabidopsis* seedlings under EGTA treatment. First, we compared the phosphoproteins and glycoproteins identified from the enriched fractions to the total protein abundance in the global proteome (Figure 6A). Notably, phosphorylation sites were predominantly associated with higher-abundance proteins, whereas N-glycosylation was often observed in lower-abundance proteins. Despite this trend, over half of the phosphoproteins were not detected in the global proteome data set, while approximately 70% of N-glycoproteins were present (Figure 6B; Table S4A,B). Furthermore, the overlap between phosphoproteome and N-glycoproteome was less than 1%, consistent with our previous findings.^34^

**Figure 6:**
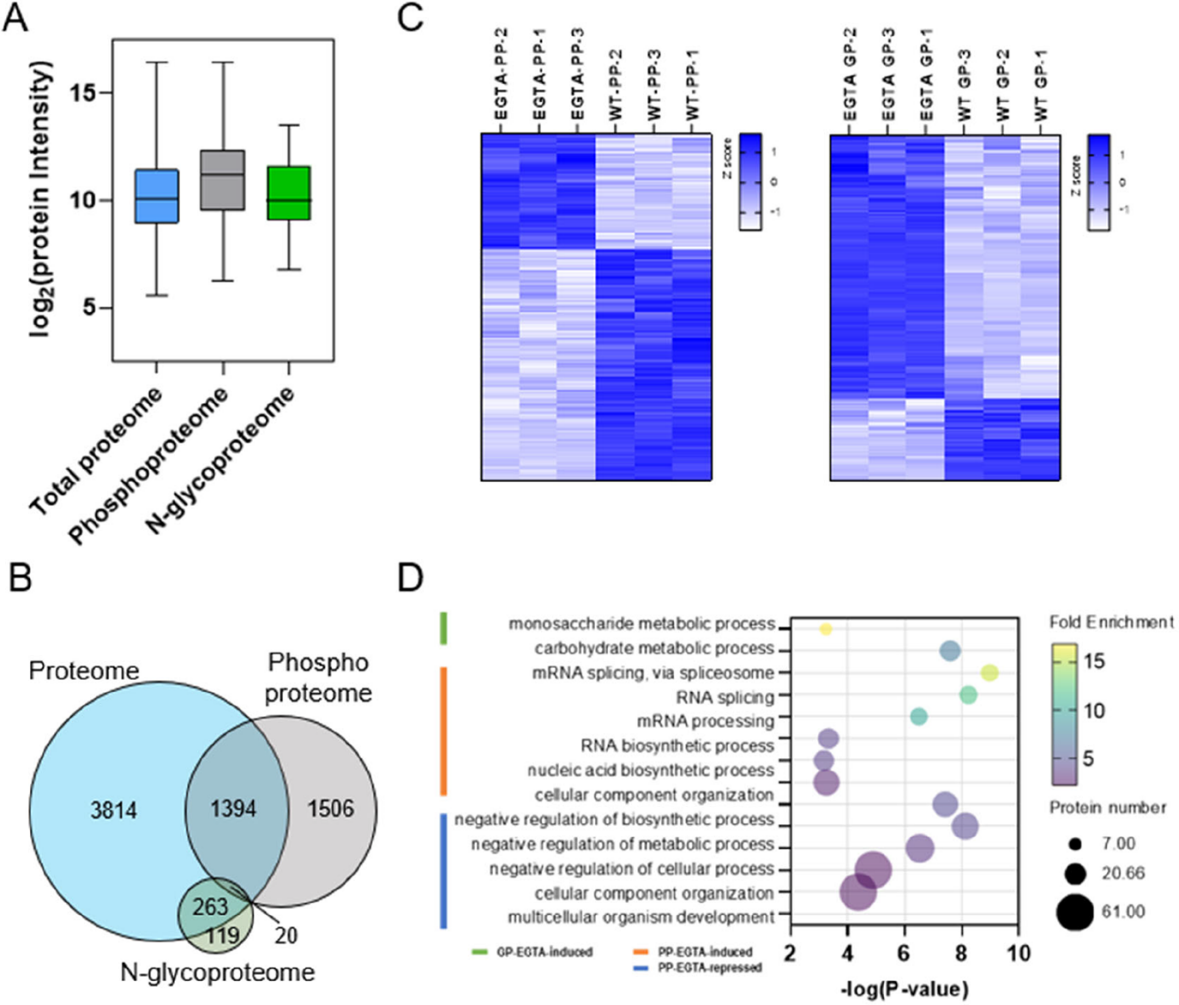
Analysis of the impact of calcium deprivation on the *Arabidopsis* phosphoproteome and N-glycoproteome using IMAC-HILIC tip. (A) Comparison of relative protein abundance distributions in the global proteome for proteins identified in the PP and GP fractions. (B) Venn diagram showing the overlap of proteins identified in the global proteome, phosphoproteome, and N-glycoproteome fractions. (C) Heatmaps depicting hierarchical clustering of quantifiable phosphorylation sites and N-glycopeptides significantly altered by EGTA treatment. (D) Selected GO Biological Process terms enriched among phosphoproteins and N-glycoproteins that were significantly modulated under EGTA treatment.

Second, distinct patterns of response were observed for the N-glycoproteome and phosphoproteome following EGTA. More N-glycopeptides were detected overall under EGTA treatment, with a higher fraction exhibiting increased abundance (Figures S6A and 6C; Table S4C). By contrast, the number of identified phosphopeptides decreased and more down-regulated phosphopeptides after EGTA treatment (Figures S6B and 6C; Table S4D). These findings suggest divergent roles for N-glycosylation and phosphorylation in mediating calcium deprivation responses. Third, GO analyses further revealed distinct functional roles for the phosphoproteins and N-glycoproteins responding to EGTA treatment (Figure 6D; Table S5). For example, proteins involved in mRNA processing (*P* = 3.2e-7) and RNA splicing (*P* = 1.0e-9) are significantly enriched among EGTA-induced phosphoproteins, implying a role for the phosphoprotein in reprogramming gene expression under calcium-deficient conditions. Meanwhile, the N-glycoproteins that increased under EGTA treatment were linked to monosaccharide (*P* = 0.000576) and carbohydrate metabolic processes (*P* = 2.6e-8), suggesting a glycosylation-mediated adjustment of metabolic pathways that are crucial for stress adaptation. Collectively, these findings demonstrate how dual PTM profiling via IMAC-HILIC can uncover unique regulatory insights not captured by analyzing the phosphoproteome or N-glycoproteome alone.

## Conclusions

Incorporating readily available IMAC and HILIC materials into a single-tip format enables multidimensional analyses of crosstalk between plant phosphoproteome and N-glycoproteome. Here, we optimized the key IMAC-HILIC parameter—the HILIC elution buffer—to minimize phosphopeptide coelution in the GP fraction. We found that 1% AA efficiently releases N-glycopeptides from HILIC while preserving phosphopeptides on Fe^3+^-IMAC, as evidenced by the minimal overlap between the PP and GP fractions. Benchmarking this single-tip approach against a tandem-tip enrichment method revealed a superior reproducibility and quantitative accuracy. Finally, we demonstrated the applicability of this method for investigating signal transduction in *Arabidopsis* under calcium deprivation, underscoring its potential for dissecting complex plant regulatory networks.

It is noteworthy that in dual-mode enrichment systems utilizing immobilized titanium ions for phosphopeptide capture, organic acids such as FA and TFA typically do not disrupt the interactions between phosphopeptides and Ti^4+^ ions due to their higher affinity compared to Fe^3+^ ions.^46,47^ Therefore, evaluating optimal elution conditions for each enrichment material is critical for maximizing separation efficiency. One current limitation of the IMAC-HILIC tip is reduced phosphopeptide recovery compared to that of the tandem-tip format. This issue arises from the use of a C8 disk as the frit, which necessitates an organic solvent (e.g., ACN) for phosphopeptide elution, thereby requiring additional steps, such as tube collection and lyophilization, that can lead to sample loss. Future optimization should include evaluating alternative frit materials that permit the aqueous elution of phosphopeptides while maintaining high reproducibility.

## Supplementary Material













## Data Availability

The MS proteomics data have been deposited to the ProteomeXchange Consortium^48^ via the PRIDE partner repository with the data set identifier PXD061247.

## Notes

Representation of the IMAC-HILIC tip; assessment of AA, TFA, and FA in eluting phosphopeptides from Fe^3+^-IMAC; efficiency of AA, TFA, and FA in eluting N-glycopeptides from HILIC; overlap analysis of identified N-glycopeptides and phosphopeptides in GP and PP fractions; comparison of identification coverage and peptide characteristics between the IMAC-HILIC tip and TIMAHAC; and IMAC-HILIC tip performance in analyzing EGTA-treated phosphoproteome and N-glycoproteome (PDF)
All phosphopeptides identified in the first elution using 1% AA; all phosphopeptides identified in the first elution using 0.5% TFA; all phosphopeptides identified in the first elution using 0.5% FA; all phosphopeptides identified in the second elution following the first elution with 1% AA; all phosphopeptides identified in the second elution following the first elution with 0.5% TFA; and all phosphopeptides identified in the second elution following the first elution with 0.5% FA (XLSX)
All N-glycopeptides identified with 1% AA elution; all N-glycopeptides identified with 0.5% TFA elution; and all N-glycopeptides identified with 0.5% FA elution (XLSX)
All phosphopeptides identified in the PP fraction using the IMAC-HILIC tip; all phosphopeptides identified in the PP fraction using the TIMAHAC method; all N-glycopeptides identified in the GP fraction using the IMAC-HILIC tip; and all N-glycopeptides identified in the GP fraction using the TIMAHAC method (XLSX)
All phosphopeptides identified in control and EGTA-treated samples; all N-glycopeptides identified in control and EGTA-treated samples; phosphorylation sites significantly altered by EGTA treatment; and N-glycopeptides significantly altered by EGTA treatment (XLSX)
GO analysis of phosphoproteins induced by EGTA treatment; GO analysis of phosphoproteins repressed by EGTA treatment; GO analysis of glycoproteins induced by EGTA treatment; and GO analysis of glycoproteins repressed by EGTA treatment (XLSX)
