# Correction to “Integrated Single-Tip IMAC-HILIC Enables Simultaneous Analysis of Plant Phosphoproteomics and N‑Glycoproteomics”

**DOI:** 10.1021/acs.jproteome.6c00035

**Published:** 2026-03-10

**Authors:** Chin-Wen Chen, Ting-An Chen, Pei-Yi Lin, Shu-Yu Lin, Chuan-Chih Hsu

**Affiliations:** ‡ Institute of Plant and Microbial Biology, 38017 Academia Sinica, Taipei 115201, Taiwan; § Academia Sinica Common Mass Spectrometry Facilities for Proteomics and Protein Modification Analysis, 38017 Academia Sinica, Taipei 115201, Taiwan

The affiliation for authors Chin-Wen Chen, Ting-An Chen, Pei-Yi Lin, and Chuan-Chih Hsu was listed incorrectly in the original publication. The word “**Institution**” has been corrected to “**Institute**”.

The correct affiliations for all authors are listed.

