# Supplementary material for: Integrated Single-Tip IMAC-HILIC Enables Simultaneous Analysis of Plant Phosphoproteomics and N‑Glycoproteomics
Source: J Proteome Res. 2025 Jun 23;24(7):3560–8. doi: 10.1021/acs.jproteome.5c00185 (PMC12235695; doi:10.1021/acs.jproteome.5c00185)
Supplement: Supplementary file 1 [file pr5c00185_si_001.pdf]

# Supporting Information

**Integrated Single-Tip IMAC-HILIC Enables Simultaneous Analysis of Plant Phosphoproteomics and N-glycoproteomics**

Chin-Wen Chen[1,#], Ting-An Chen[1,#], Pei-Yi Lin[1], Shu-Yu Lin[2], Chuan-Chih Hsu[1,*]

[1]Institution of Plant and Microbial Biology, Academia Sinica, Taipei 115201, Taiwan

[2]Academia Sinica Common Mass Spectrometry Facilities for Proteomics and Protein Modification Analysis, Academia Sinica, Taipei 115201, Taiwan

[#]Both authors contribute equally to this work.

*Corresponding author:

Chuan-Chih Hsu, Phone: +886-2787-1157; Email: cchsu@gate.sinica.edu.tw

## Table of Contents

**Supporting Experimental Section**

Chemicals and Materials ........ S-3
Plant Culture and Treatment ........ S-3
Protein Lysis and Digestion ........ S-3
IMAC Enrichment ........ S-4
HILIC Enrichment ........ S-4
LC-MS/MS Analysis ........ S-5

**Supplemental Figures**

Figure S1. Representation of the IMAC-HILIC tip. ........ S-6
Figure S2. Assessment of AA, TFA, and FA in eluting phosphopeptides from $Fe^{3+}$-IMAC. ........ S-7
Figure S3. Efficiency of AA, TFA, and FA in eluting N-glycopeptides from HILIC. S-8
Figure S4. Overlap analysis of identified N-glycopeptides and phosphopeptides in GP and PP fractions. ........ S-9
Figure S5. Comparison of identification coverage and peptide signals between IMAC-HILIC tip and TIMAHAC. ........ S-10
Figure S6. IMAC-HILIC tip performance in analyzing EGTA-treated phosphoproteome and N-glycoproteome........ S-11

**Supplemental Tables**

Table S1. Phosphopeptides identifications under different elution schemes.
Table S2. N-glycopeptide identifications under different elution conditions.
Table S3. Phosphopeptides and N-glycopeptides identified using the IMAC-HILIC tip or TIMAHAC approach.
Table S4. Overview of phosphopeptide and N-glycopeptide identifications in control and EGTA-treated samples.
Table S5. GO analysis of phosphoproteins and glycoproteins that were significantly altered following EGTA treatment.

## Supporting Experimental Section

### Chemicals and Materials

Urea, tris(2-carboxyethyl) phosphine hydrochloride (TCEP), 2-chloroacetamide (CAA), triethylammonium bicarbonate (TEAB), iron chloride, trifluoroacetic acid (TFA), ethylenediaminetetraacetic acid (EDTA), and sodium dodecyl sulfate (SDS) were purchased from Sigma-Aldrich. Phosphoric acid (PA) was purchased from Honeywell Fluka. Methanol (MeOH), formic acid (FA) and SeQuant ZIC-pHILIC column were purchased from Merck. Acetic acid (AA), acetonitrile (ACN), and ammonia phosphate ($NH_4H_2PO_4$) were purchased from J.T.Baker. Ni-NTA silica beads were purchased from Qiagen. Evotips were purchased from Evosep. S-Trap micro columns were purchased from ProtiFi (Huntington, NY). Empore C8 extraction disks were purchased from 3M. MS grade Lys-C (lysyl endopeptidase) was purchased from FUJIFILM Wako. Sequencing-grade modified trypsin was purchased from Promega. Water was obtained from a Millipore Milli-Q system.

### Plant Culture and Treatment

Arabidopsis thaliana (Columbia-0) seeds were sterilized and sown on half-strength Murashige and Skoog (1/2 MS) medium, then at a 4 °C for 2 days to synchronize germination. For workflow optimization, seedlings were grown vertically on 1/2 MS plates for 14 days prior to harvesting. For ethylene glycol-bis(2-aminoethylether)-N,N,N',N'-tetraacetic acid (EGTA) treatment experiments, seedlings were grown vertically on 1/2 MS plates for 7 days, transferred to a conical flask containing 1/2 MS medium for a 3-day acclimation period, and then exposed to either 1/2 MS medium (control) or 20 mM EGTA (pH 8.0) for 2 h.

### Protein Lysis and Digestion

Protein lysis and enzymatic digestion were conducted as described previously, with minor modifications. Briefly, Arabidopsis seedlings were flash-frozen in liquid nitrogen and pulverized into a fine powder using a mortar and pestle. The resulting powder was lysed in 8 M urea prepared in 50 mM triethylammonium bicarbonate (TEAB) and transferred to a 1.7-mL microcentrifuge tube. Samples were sonicated (10 cycles of 10 s each) and then centrifuged at 14,000*g* for 10 min at room temperature to remove debris. Protein concentrations in the supernatant were determined using the bicinchoninic acid assay (Thermo Fisher Scientific, Waltham, MA).

Next, 100 µg of protein (in the lysis buffer) was combined with an equal volume of 10% (v/v) sodium dodecyl sulfate (SDS) in 50 mM TEAB. Proteins were reduced and alkylated using 10 mM Tris(2-carboxyethyl) phosphine hydrochloride (TCEP) and 40 mM 2-chloroacetamide (CAA) at 45 °C for 15 min. Phosphoric acid was then added

to achieve a final concentration of 5.5% (v/v), followed by six volumes of binding buffer (90% v/v methanol in 100 mM TEAB). After gentle vortexing, the sample was loaded onto an S-Trap micro column and centrifuged at 4000*g* for 1 min to remove the flow-through. The column was washed three times with 150 µl of binding buffer. Proteolytic digestion was performed by adding 20 µl of digestion solution containing 1 unit of Lys-C and 2 µg of trypsin in 50 mM TEAB and incubated the column at 47 °C for 2 h. Finally, peptides were eluted with 150 µL of 1% (v/v) trifluoroacetic acid (TFA) in 80% (v/v) acetonitrile (ACN) and directly loaded onto $Fe^{3+}$-IMAC, an HILIC, or IMAC-HILIC tips using the tandem tip format via centrifugation, as appropriate for subsequent analyses.

**IMAC Enrichment**

Phosphopeptide enrichment was performed using an established $Fe^{3+}$-IMAC protocol with minor modifications. Briefly, an in-house IMAC tip was generated by placing a 20-µm polypropylene frit disk at the distal end of a 200-µL pipette tip, then packing the tip with 5 mg of $Ni^{2+}$-NTA silica beads obtained from a Ni-NTA spin column. The assembled tip was inserted into a 2-mL microcentrifuge tube and loaded with 100 mM ethylenediaminetetraacetic acid (EDTA) (200*g*, 2 min) to remove $Ni^{2+}$ ions. The beads were subsequently activated with 100 mM $FeCl_3$ (200*g*, 2 min) and equilibrated with 1% (v/v) TFA, 80% (v/v) ACN (200*g*, 2 min).

Tryptic peptides, eluted from the S-Trap microcolumn with 1% (v/v) TFA and 80% (v/v) ACN, were directly loaded onto the IMAC tip (200g, 2 min). Following two washes with 100 µL of 1% (v/v) TFA and 80% (v/v) ACN (200*g*, 2 min per wash), and a single wash with 1% (v/v) acetic acid (AA) (pH 3.0; 200*g*, 2 min), the IMAC tip was placed into an activated Evotip. Phosphopeptides were then eluted from the IMAC beads into the Evotip using 200 mM $NH_4H_2PO_4$ (800*g*, 5 min) and subsequently analyzed via LC-MS/MS on an Evosep One system.

**HILIC Enrichment**

The ZIC-HILIC silica bead was obtained from a SeQuant ZIC-pHILIC column. A HILIC tip was prepared by affixing a C8 extraction disk at the distal end of a 200-µL pipette tip and placing the tip into a 2-mL microcentrifuge tube. The HILIC beads were activated with 200 µL of 1% (v/v) TFA before loading into the tip. The C8 disk was activated with 20 µL of 1% (v/v) TFA, and 80% (v/v) ACN (centrifuged at 1000*g*, 5 min), and subsequently packed with 10 mg of HILIC silica beads. The tip was washed once with 1% (v/v) TFA (1000*g*, 5 min) and once with 1% (v/v) TFA, and 80% (v/v) ACN (1000*g*, 5 min).

Tryptic peptides, eluted from the S-Trap microcolumn using 1% (v/v) TFA and 80% (v/v) ACN, were loaded into the HILIC tip via tandem-tip centrifugation at 1000*g* for 5 min. The tip was subsequently washed three times with 1% (v/v) TFA in 80% (v/v) ACN (1000*g*, 5 min each). Next, the HILIC tip was inserted into an activated Evotip, and the N-glycopeptides were eluted into the Evotip using 0.5% (v/v) formic acid (FA) (800*g*, 5 min). Finally, the Evotip was placed into an Evosep One LC system for LC-MS/MS analysis.

**LC-MS/MS Analysis**

The sample was loaded onto Evotips Pure and analyzed on an Evosep One LC system (EvoSep) coupled to a timsTOF HT mass spectrometer (Bruker Daltonics). Phosphopeptides and N-glycopeptides were separated using the 30 SPD method on an Evosep Performance column (15 cm × 150 µm ID, 1.5 µm, EV1137; EvoSep), maintained at 40 °C within a Captive Spray ion source (Bruker Daltonics), equipped with a 20 µm emitter (ZDV Sprayer 20, Bruker Daltonics).

Data acquisition was carried out with timsControl 4.0 (Bruker Daltonics) in data dependent acquisition (DDA) parallel accumulation-serial fragmentation (PASEF) mode. The trapped ion mobility spectrometry (TIMS) dimension was calibrated using the Agilent ESI LC/MS tuning mix in positive mode with the following m/z values and 1/K0 values: (622.0289, 0.9848 Vs/$cm^2$), (922.0097, 1.1895 Vs/$cm^2$), and (1221.9906, 1.3820 Vs/$cm^2$). For phosphopeptide analysis, 10 PASEF/MSMS scans were acquired per cycle over an *m/z* range of 100–1700 and an ion mobility (IM) of 0.6–1.6 V s/$cm^2$. The accumulation and ramp time were both set to 100 ms. The capillary voltage was maintained at 1550 V, and the ion mobility-dependent collision energy (CE) was linearly ramped from 20 eV at 0.60 V s/$cm^2$ to 59 eV at 1.6 V s/$cm^2$. Singly charged precursors were excluded based on a polygon filter in the *m/z*-IM plane, and precursors signals above an intensity threshold of 2500 were selected for fragmentation. Isolation windows were 2 Thomson (Th) for precursor $m/z < 700$ and 3 Th for $m/z > 800$. Precursors were actively excluded for 0.4 min after reaching a target intensity of 20,000. For glycopeptides analysis, the *m/z* range was set to 100–2800, and a stepping CE was used to enhance N-glycopeptide dissociation. Each stepping PASEF MS/MS frame consisted of two merged TIMS scans acquired at lower (32–64 eV) and higher (40–100 eV) CEs. Six PASEF MS/MS scans were triggered per cycle, each with a target intensity of 40,000, and the IM scan range was set to 0.68–1.66 Vs/$cm^2$.

## Supplemental Figures

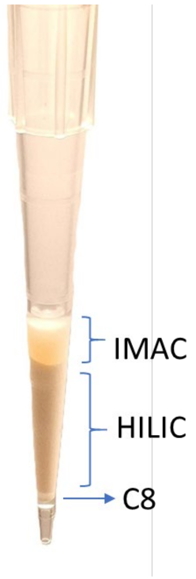


**Figure S1. Representation of the IMAC-HILIC tip.** A C8 disk frit was placed at the distal end of a 200-µL pipette tip, followed by sequential packing of HILIC beads and then IMAC beads through centrifugation

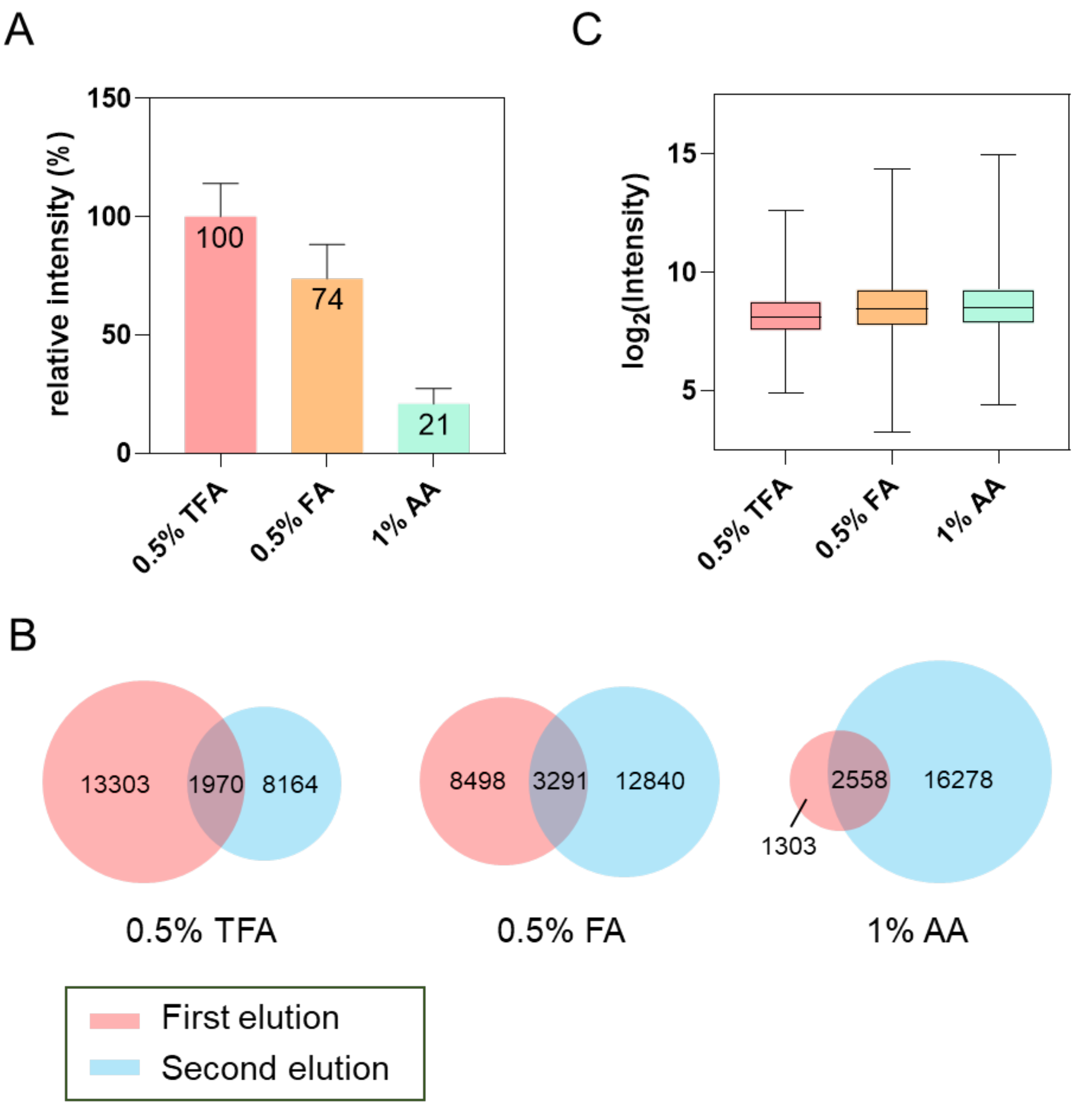


**Figure S2. Assessment of AA, TFA, and FA in eluting phosphopeptides from $Fe^{3+}$-IMAC.** (A) Accumulated XIC area of monophosphorylated peptides after elution with AA, TFA, and FA, with the total intensity for TFA set to 100%. (B) Venn diagram illustrating the overlap of phosphopeptides identified between the first and second elution steps. (C) Boxplot showing the distribution of log2 MS1 intensities for monophosphorylated peptides identified in the second elution, following the first elution with AA, TFA, and FA.

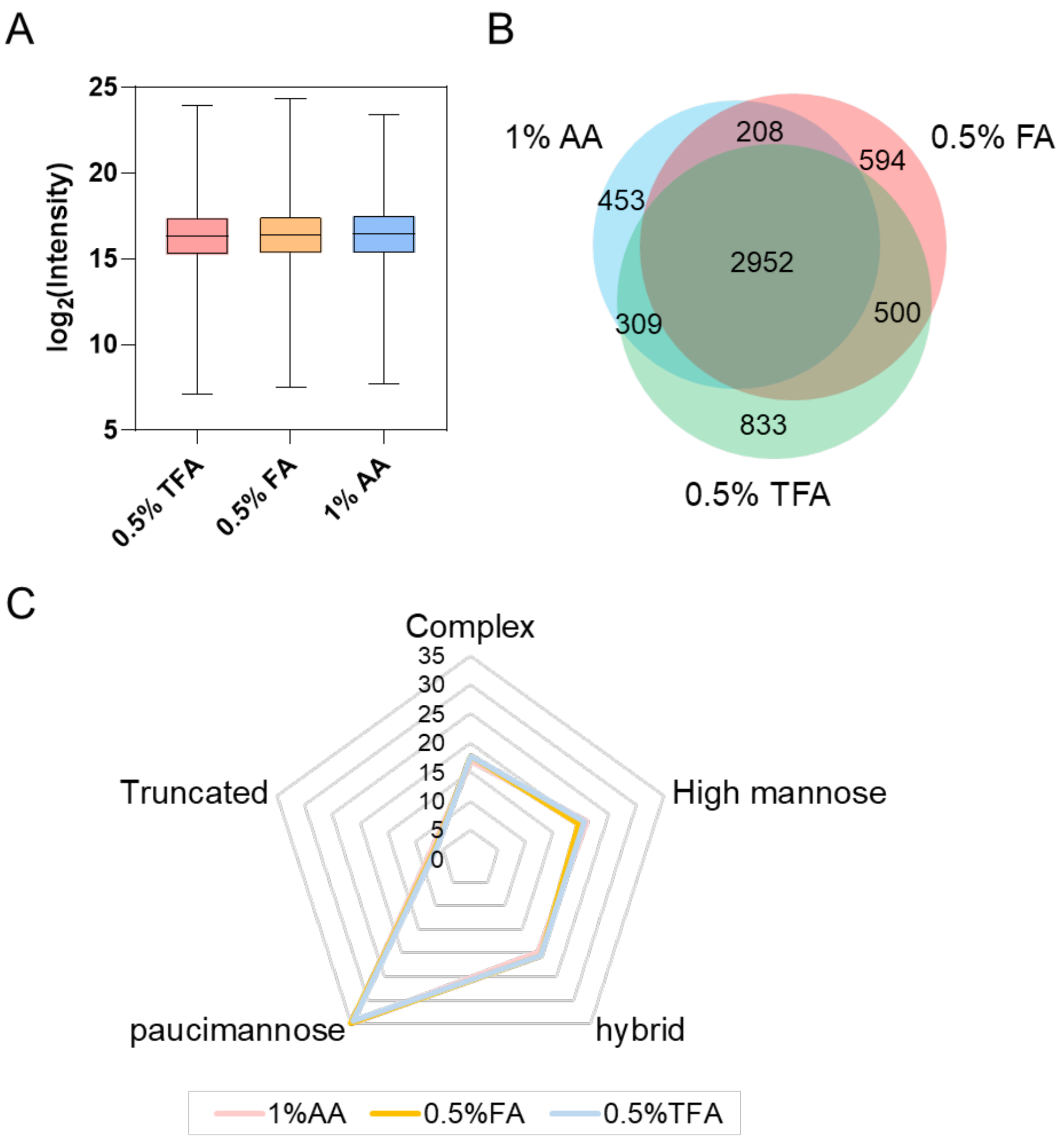


**Figure S3. Efficiency of AA, TFA, and FA in eluting N-glycopeptides from HILIC.** (A) Boxplot illustrating the distribution of $log_2$ MS1 intensities of the identified N-glycopeptides under the three elution conditions. (B) Venn diagram showing the overlap of N-glycopeptides identified from elutions with 1% AA, 0.5% TFA, and 0.5% FA. (C) Radar plot depicting the distribution of five N-glycan categories among the unique N-glycoforms identified under each elution condition.

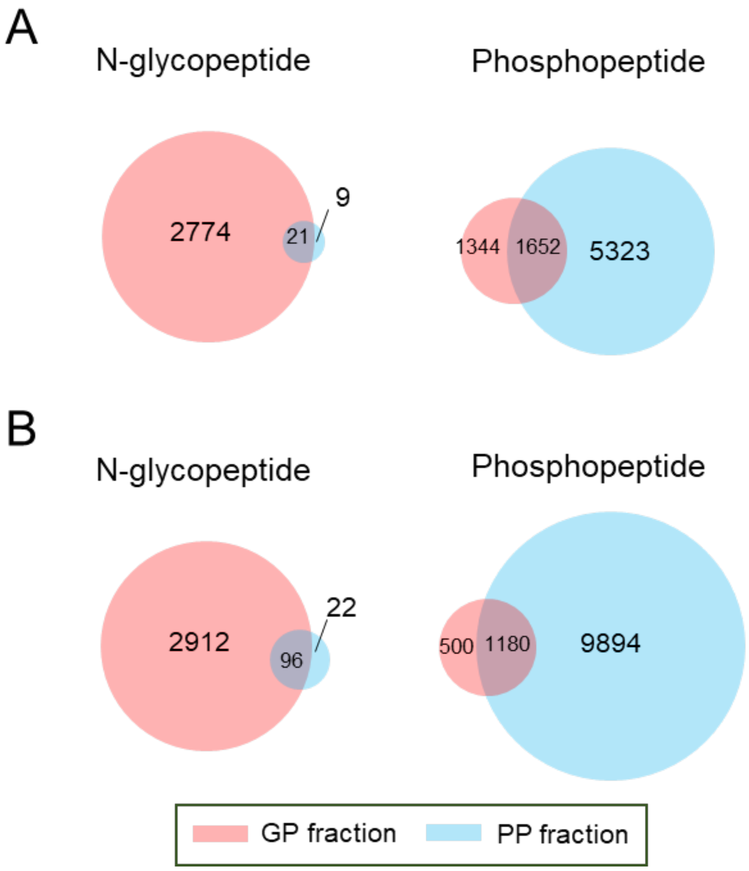


**Figure S4. Overlap analysis of identified N-glycopeptides and phosphopeptides in GP and PP fractions.** (A) GP fraction eluted using TFA. (B) GP fraction eluted using FA.

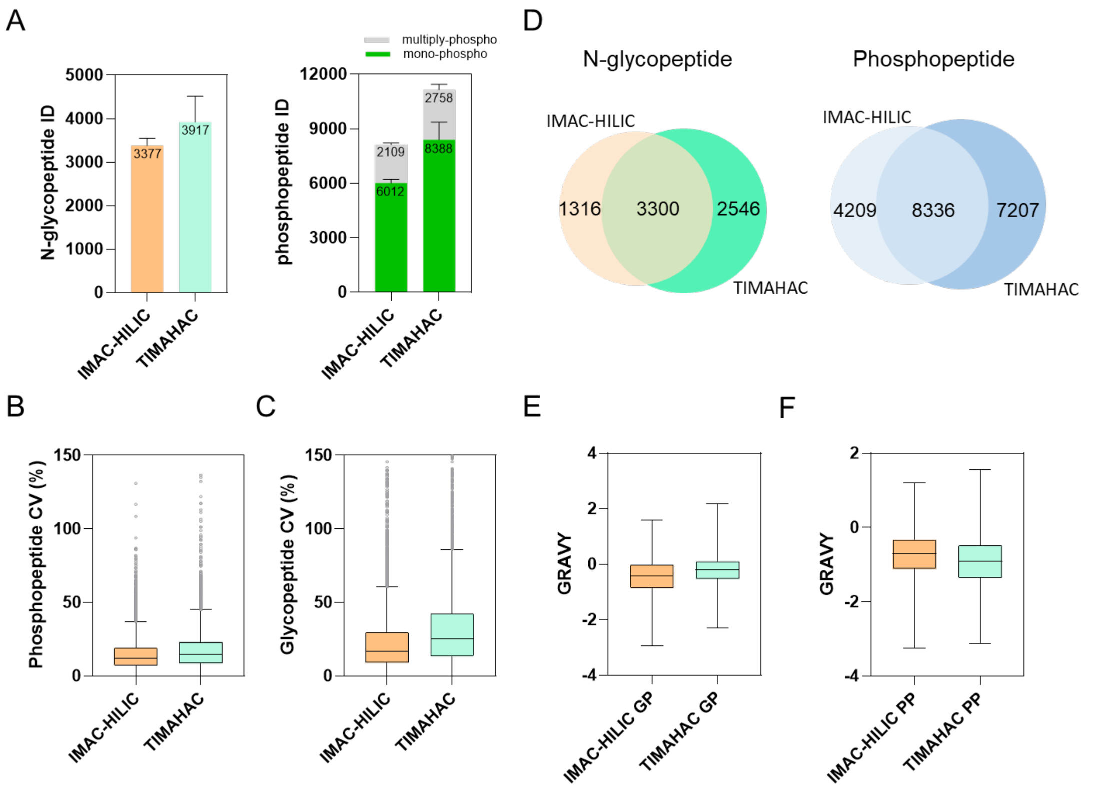


**Figure S5. Comparison of identification coverage and peptide characteristics between IMAC-HILIC tip and TIMAHAC.** (A) Number of N-glycopeptides (left) and phosphopeptides (right) identified with IMAC-HILIC and TIMAHAC. (B) Distribution of CV values for phosphopeptides identified in the PP fraction using both methods. (C) Distribution of CV values for N-glycopeptides identified in the GP fraction using both methods. (D) Venn diagram illustrating the overlap of N-glycopeptides and phosphopeptides identified by IMAC-HILIC and TIMAHAC. (E) Distribution of GRAVY values for N-glycopeptides in the GP fraction. (F) Distribution of GRAVY values for phosphopeptides in the PP fraction.

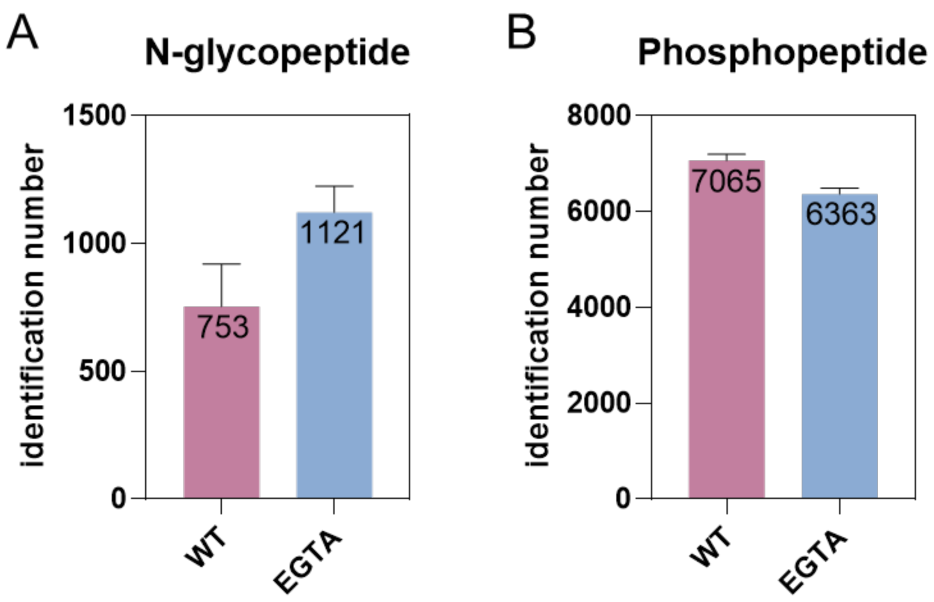


**Figure S6. IMAC-HILIC tip performance in analyzing EGTA-treated N-glycoproteome and phosphoproteome.** (A) Number of N-glycopeptides identified in control and EGTA-treated samples. (B) Number of phosphopeptides identified in control and EGTA-treated samples.
